# Fabrication of 2D Hetero-Complexes With Nucleic-Acid-Base Adenine and Fatty-Acid Stearic Acid at Liquid/Solid Interface

**DOI:** 10.3389/fchem.2019.00513

**Published:** 2019-07-25

**Authors:** Huiling Zhao, Qian Yang, Zegao Wang, Hang Zhao, Bo Liu, Qianming Chen, Mingdong Dong

**Affiliations:** ^1^School of Physics and Electronics, Henan University, Kaifeng, China; ^2^Interdisciplinary Nanoscience Centre (iNANO), Sino-Danish Center for Education and Research (SDC), Aarhus University, Aarhus, Denmark; ^3^College of Materials Science and Engineering, Sichuan University, Chengdu, China; ^4^State Key Laboratory of Oral Diseases, National Clinical Research Center for Oral Diseases, West China Hospital of Stomatology, Sichuan University, Chengdu, China

**Keywords:** scanning tunneling microscopy (STM), supramolecular self-assembly, non-covalent interaction, hydrogen bond, building block

## Abstract

Designing and fabricating hetero-complexes composed of organic and biological compounds had become an exciting area referring to biological recognition, molecular devices etc. Here, hydrogen-bonded complex of nucleic-acid-base (adenine, A) and fatty-acid (stearic acid, SA) was designed, fabricated and investigated at liquid/solid interface. The interesting striped-shaped structure composed of SA-A-SA trimers was formed after introducing adenine molecules. Meanwhile, the primary lamella-shape characteristic of the assembly of SA molecules was kept because of the collaboration of non-covalent interactions of molecule-molecule and molecule-substrate. With a series of experimental characterization and theoretical simulation, the origination of the as-prepared 2D hetero-complexes was gradually exhibited from the assembled structures of two building blocks of stearic acid and adenine. Our study provides a blueprint for designing additional multi-component complexes based on the existing molecular assembled architectures.

## Introduction

Hetero-complexes composed of chemical and biological compounds have emerged as a novel category of molecule-based materials, which exhibit tremendous potential, used as regenerative medicine (Hudalla et al., 2014; Webber et al., 2016) and molecular devices in advanced technologies (Horiuchi et al., 2005; Horiuchi and Tokura, 2008; Cademartiri and Bishop, 2015; Wang et al., 2018a). In order to achieve specific functionality, controlling their lateral assembly and spatial assembled behaviors of building blocks is the vital issue during the fabrication procedure for the desired hetero-complexes. Supramolecular assembly (SMA), being one versatile bottom-up approach, provides a simple means to interlink single or multiple components together into functional ensembles or well-ordered architectures with different dimensions (Whitesides et al., 1991; Huang et al., 2011; Claridge et al., 2013; Liu et al., 2013; Wang et al., 2018b). In current SMA fields, there are masses of mono-component assemblies sophisticatedly prepared by choosing appropriate building blocks, solvents, and other experimental conditions. On the contrary, fabricating hetero-complexes is still one big challenge because two problems occur occasionally during binary or multiple assembly systems (Tahara et al., 2015; Banerjee et al., 2016; Ibenskas et al., 2016). One is the phase separation at nanoscale, and the other one is the formation of randomly mixed monolayers, both of which go against the successful utilization of the heterojunction materials for advanced technological applications.

It is well-acknowledged that all supramolecular assemblies are the collaborative results of multiple non-covalent interactions of molecule-molecule (Bilbao et al., 2016), molecule-substrate (MacLeod et al., 2015) and molecule-solvent (Chen et al., 2010). Therefore, several experimental factors such as the characteristics of building blocks, intrinsic properties of substrate surface and conditions of assembly interface, had been explored on purpose during the assembly processes. In addition, pre-fabricated supramolecular assembles with mono-component have been gradually utilized as advisable model systems or available templates for the design of hetero-component assemblies (Guo et al., 2013; Ghijsens et al., 2015; Plas et al., 2016). For example, long-chain hydrocarbons including n-alkanes, n-alkanols, n-fatty acids, and their substituents had been used as model systems to study the effect of non-covalent interactions on the formation of molecular assembled patterns. Based on the self-assembled monolayer of 2,6,10-tri-carboxydecyloxy-3,7,11-triundecyloxy triphenylene (sym-TTT), a guest molecule such as phthalcyanine (ZnPC) and melamine had been efficiently introduced to construct the hetero-component assemblies of ZnPc/sym-TTT (Li et al., 2010) and sym-TTT/melamine (Li et al., 2012). Then these pre-prepared networks of sym-TTT/melamine could be further used to capture a second guest metallic ion of Fe^3+^. The trimers of sym-TTT/melamine/Fe^3+^ were successfully formed by this step-by-step multi-component self-assembly strategy finally.

In order to explore more effective route for fabricating more hetero-component assembles, here we try to design one simple strategy to assemble a binary-component hetero-complex composed of organic compounds and biological compounds. In this work, we selected two kinds of molecular solution at room temperature, and investigated their assemblies of these building blocks at liquid/solid (1-phenyloctane/graphite) interface. With a series of experimental characterization and theoretical simulation, the assembled structures of two building blocks were gradually exhibited, and the detailed intermolecular interactions of the as-prepared two-dimensional (2D) hetero-complexes was revealed particularly.

## Experimental

### Materials and Sample Preparation

Two kinds of molecules, adenine (A) with 98% pure and stearic acid (SA) with ≥98.5% pure, were bought from Sigma-Aldrich Corporation. At room temperature, these powders were dissolved into 1-phenyloctane solvent (99% pure, Sigma-Aldrich Inc.) to produce A- and SA- saturated solutions, respectively. Then A-saturated solution was mixed with SA-saturated solution with the volume ratio of 1:1. After dropping their totally mixed solution onto a freshly-cleaved substrate surface, scanning tunneling microscopy (STM) characterization were carried out under ambient condition.

### Characterization Method

All STM characterization was performed at the liquid/solid (1-phenyloctane/graphite) interface using a MultiMode SPM system with a Nanoscope IIIa controller (Veeco Instruments Inc., Santa Barbara, CA). STM tips were mechanically cut from a piece of Pt/Ir (80/20) wire with 0.25 mm diameter (Nanoscience Instruments Inc., Phoenix, USA), and tested on freshly-cleaved highly oriented pyrolytic graphite (HOPG, grades ZYA and ZYB, Advanced Ceramics Inc., Cleveland, OH and NT-MDT, respectively) surfaces. All STM images were recorded in constant current mode, and under various tunneling conditions with tunneling currents (0.5~1.0 nA) and positive sample voltages (0.5~0.8 V).

### Image Analysis and Theory Simulation

The images used in this paper were subsequently processed using the correlation averaging method provided by the Scanning Probe Image Processor software (SPIP, Image Metrology A/S, Lyngsø, Denmark). A maximum of 10 averages was adopted to improve the signal-to-noise ratio and to maintain the characteristic structural features of the molecular self-assembled patterns. In addition, all theoretical models for adenine and stearic acid were built by Dmol3 module of the Material Studio software, and the substrate was not included because of the performance limitation of the used computing cluster.

## Results and Discussion

**Figure 1:**
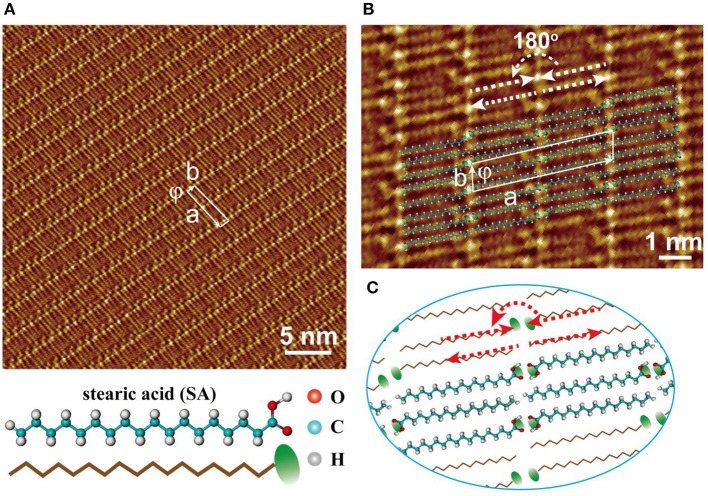
**(A)** STM image for the self-assembled pattern formed by stearic acids. **(B)** The high-resolution STM image of self-assembled stearic acids with molecular model overlapped. **(C)** The molecular model corresponding to the assembled patterns shown in **(B)**.

Various assembled patterns of long-chain hydrocarbons and their derivatives have been systematically studied and further used as molecular templates in many works (McGonigal et al., 1990; Rabe and Buchholz, 1991; Yablon et al., 2002; Wintgens et al., 2003). For example, stearic acid, one of widely-adopted building blocks, is easily assembled on freshly-cleaved HOPG surface. Because stearic acid consists of functional carboxylic group and alkane backbone (seeing the chemical structure of stearic acid in Figure 1), lamellar structure can be formed under the cooperation of two main kinds of non-covalent interactions. One kind is the molecule-molecule interaction forming hydrogen bonds between neighboring stearic acid molecules. The other kind is the molecule-substrate interaction because the alkane backbone of stearic acid molecular chains has a good match with the lattice periodicity of graphite surface (Li et al., 2011). Being drived by these molecular non-vovalent interactions, one kind of assembled patterns is formed by stearic acid molecules as shown as Figures 1A,B, respectively.

In order to investigate the molecular interactions between stearic acid, the high-resolution STM image of self-assembled pattern with molecular models overlapped had been also given in Figure 1B. The unit cell was drawn out as indicated by a white rectangle in Figure 1B, and its parameters are a = 4.43 ± 0.20 nm, b = 0.82 ± 0.20 nm, and ϕ = 80.0 ± 2°. According to the odd-even effect of saturated all-trans carboxylic acids, stearic acid with even-numbered all-trans carbon atoms tends to form an enantiomer structure on HOPG surface. In each lamella, two parallel stearic acid molecules were packed together with head-to-head configuration through –COOH interacting with –COOH between adjacent stearic acids, and tail-to-tail configurations through –CH_3_ interacting with –CH_3_ in the adjacent one lamella. In two adjacent lamellas, two neighboring stearic acid molecules can also form a similar head-to-tail configuration existed with head-to-head state. Under these dense intermolecular hydrogen bonding and the supplementary interaction of molecule-substrate, a large uniform and flat domain was easily assembled with the lamella characteristics presented by stearic acid molecules. Because of the different intrinsic electronic properties of carboxylic group and alkane backbone, they always give different current tunneling efficiencies. That is, the bright dots presented the location of carboxylic groups in STM images for the assembled pattern of stearic acid. These results indicate that the self-assembled patterns formed at liquid/solid interface are various or diversiform since liquid environment can supply a more complex thermodynamic condition.

According to many previous works, it has been realized that understanding the experimental factors of molecular assembly and the formation mechanism of SA assembled structures as well as other hydrocarbon substituents, provides essential knowledge for further constructing molecular architectures (Hauptmann et al., 2013; Hu et al., 2015; Yokoyama et al., 2015). It is obvious that the subtle differences of chemical structures and components always bring significant structural transition in assemblies. Furthermore, these can not only highlight the complexity of surface self-assembly, but also make it possible to finely tune the assembled nanostructure. For example, using the cis- conformation of the interior –HC = CH– group had switched the extension direction of the carbon skeleton by 120° through inducing different odd-even effects in the molecular packing lamella (Tao et al., 2006). Additionally, aromatic-like rings such as phthalocyanine and porphyrin linked with different alkyl-chains, not only can change the assembled orientation of carbon skeletons, but also can supply functional groups in the assembled organic materials (Qiu et al., 2000; Lei et al., 2002; Iavicoli et al., 2010). Therefore, insightfully understanding of surface assembly of mono-component supramolecular structure and their transition under some experimental conditions, will provides valuable knowledge to explore the multi-component and hetero-complex assemblies.

**Figure 2:**
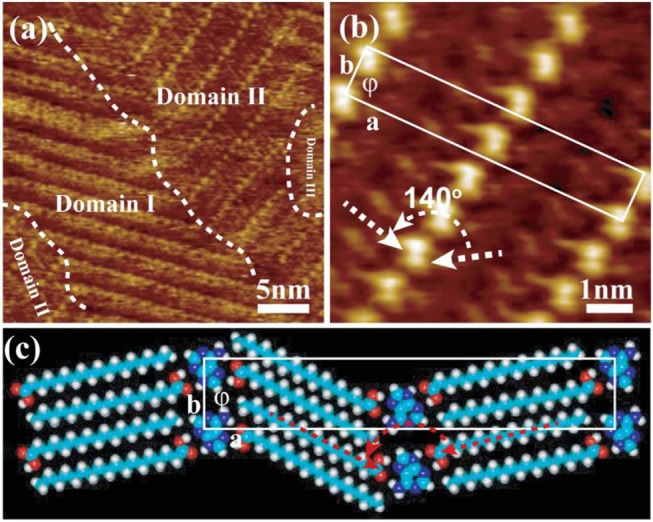
**(a,b)** STM images for the well-ordered hetero-structure (SA-A-SA) formed by A/SA detected on 1-phenyloctane/graphite interface. **(c)** The tentative molecular model for A/SA binary assembled pattern.

Nucleic acid (NA) bases including adenine (A), cytosine (C), guanine (G), thymine (T), and uracil (U), belong to another one distinguished class of molecular building blocks and have been used extensively to construct different assembly elements for biosensors (Seeman, 2003), nanomechanical devices (Liedl et al., 2007) and numerous supramolecular architectures (Ke et al., 2014) as well. In this work, adenine was chosen and its co-adsorption behavior with the lamellar assembled structure of stearic acid was investigated in order to fabricate the bi-component complex composed of biological and chemical organic molecules. A-saturated solution was mixed with SA-saturated solution with the volume ratio of 1:1 at room temperature. After dropping their totally-blended solution onto a HOPG substrate, the STM imaging was also carried out at liquid/solid (1-phenyloctane/graphite) interface. Given as Figure 2a, three different domains can be intuitively divided out in the obtained STM image with a large scanning range. That is, the characteristic lamellar structure formed by pure SA molecules is easily distinguished out and located in the Domain I, however there is no regular pattern observed in the Domain III. Interestingly, the right-top domain (marked as domain II) and another small domain in the left-bottom were consisted of one new kind of assembled structure, where building blocks are aligned in a striped structure. It is obviously different with the lamellar structure formed by pure stearic acid molecules in Figure 1. The average width of stripes can be easily sketched out by bright rows according to the right-top section of Figure 2a, and the high-resolution STM image was also given in Figure 2b. The stripes in Figure 2b are composed of one bright row in the middle of two dark rows (on the sides) to form a sandwich-like structure. In our previous study (Liu et al., 2014), the adenine molecules could self-assemble into a stable 2D network arrangement with the determined unit cell a = 0.80 ± 0.1 nm, b = 2.20 ± 0.2 nm, and ϕ = 76.0 ± 2.3° (Figure S2). It is well-known that, the different chemical structures and the intrinsic electronic properties of building blocks in the same assembly architecture always exhibit different current tunneling efficiencies which lead to the bright-dark contrast in the STM images. Evidently, there are two different components involved in this as-prepared striped structure. It had been identified that a conjugated π-electron system such as adenine tends to give higher electron conductance than alkyl chains of alkanes, and thus it will appear brighter in the STM images (Figures 2a,b). Therefore, two dark rows in Figures 2a,b, should be attributed to the alkyl chains of stearic acids and the bright dots correspond to single adenine molecule. That is, the sandwich-liked structure composed by SA-A-SA trimers was fabricated with the moderate experimental condition. The white arrays indicate the orientation of self-assembled stearic acid molecules, where the angle is about 140° which is different with the angle (about 180°) in pure stearic acid self-assembled pattern (see Figure 1B) and that (about 110°) in pure adenine self-assembled pattern (see Figure S2A). With the rotation effect played by adenine in the middle, it is easily understood why the length of *a* parameter of SA-A-SA unit cell is shorter than the value of that in SA-SA and A-A assembled unit cells molecules added together (Table S1).

**Figure 3:**
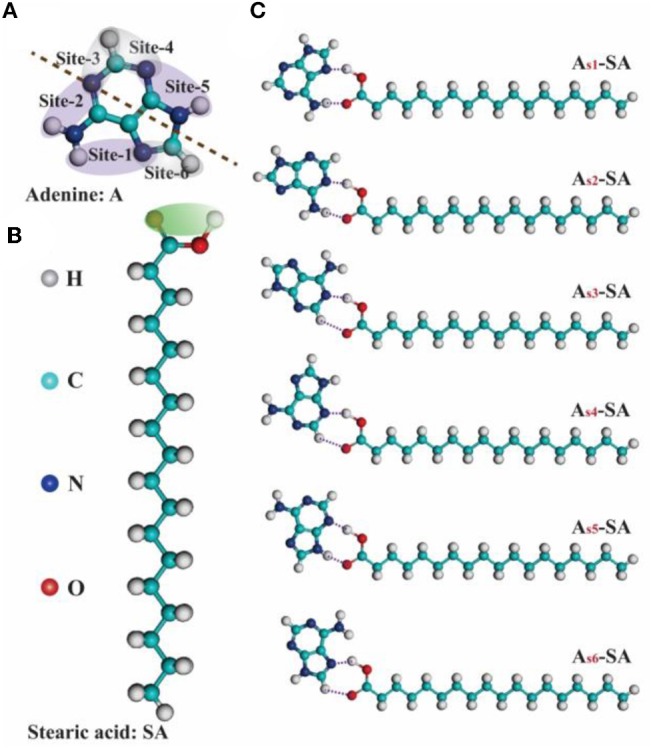
**(A,B)** The chemical structures of nucleic-acid-base adenine (A) and fatty-acid stearic acid (SA) marked with the possible reaction sites of hydrogen-bonding when they are assembled. **(C)** Six kinds of A_sx_-SA dimers (x: 1, 2, 3, 4, 5, 6) composed of one adenine and one stearic acid.

To illustrate the formation mechanism of binary assembled structure, a series of theoretical calculations were carried out. From the viewpoint of intermolecular hydrogen-bonding, the reaction sites in adenine and stearic acid, which will contribute to the formation of hydrogen-bonding, can be clearly labeled out as given Figures 3A,B. Since one adenine molecule has six hydrogen-bonding sites interacting with the carboxyl group of stearic acid, there are six kinds of A-SA dimers existing theoretically (Figure 3C). Then, the subsidiary theoretical calculation was also performed for the possible trimers formed by adenine and stearic acid molecules (see Figure S1). According to the matching of donor-acceptor pairs between N-H and O-H groups, 12 kinds of SA-_sx_A_sx_-SA trimers were built and optimized, and their corresponding binding energies were exhibited in Table 1. It is obvious that N and H atoms with different locations in adenine molecule have different electronegativity conditions, which has an important influence on the length and strength of hydrogen bonds among SA-_sx_A_sx_-SA trimers. And two higher binding energies of 1.56 and 1.52 eV were belonged to trimer SA-_s1_A_s5_-SA and SA-_s2_A_s5_-SA, respectively. This result proves that the Hoogsteen site of A is crucial in the formation of its assemblies (Moser and Dervan, 1987; Mamdouh et al., 2006). Therefore, it was indicated that a highest probability of forming trimer SA-_s1_A_s5_-SA existed when mixing A-solution with SA-solution together.

**Table 1:** The summary table for the binding energy (ΔE_BE_) of the SA-A-SA trimers shown in Figure S1.

	**Binding energy ΔE_**BE**_ (eV/Trimer)**		**Binding energy ΔE_**BE**_ (eV/Trimer)**
SA-_S1_A_S2_-SA	−1.39	SA-_S2_A_S6_-SA	−1.24
SA-_S1_A_S3_-SA	−1.20	SA-_S3_A_S4_-SA	−0.78
SA-_S1_A_S4_-SA	−1.16	SA-_S3_A_S5_-SA	−1.27
SA-_S1_A_S5_-SA	−1.56	SA-_S3_A_S6_-SA	−0.97
SA-_S1_A_S6_-SA	N/A	SA-_S4_A_S5_-SA	N/A
SA-_S2_A_S3_-SA	N/A	SA-_S4_A_S6_-SA	−0.98
SA-_S2_A_S4_-SA	−1.20	SA-_S5_A_S6_-SA	−1.29
SA-_S2_A_S5_-SA	−1.52	Trimer: SA-_SX_A_SX_-SA (X = 1,2,3,4,5,6)	

*The related theoretical calculations were carried out by using DMol3 package of Material Studio software. A, adenine; SA, stearic acid*.

According to STM image of Figure 2b, a tentative molecular model of SA-A-SA is shown in Figure 2C. In this model, trimer SA-_s1_A_s5_-SA acts as secondary building blocks with the hydrogen bonds of N-H and O-H formed by one adenine vs. two stearic acid molecules. Every adenine molecule in the middle of SA-_s1_A_s5_-SA trimer was immobilized with the same aligned direction in each bright stripe line. Meanwhile, adenine molecules in two neighboring striped lines are oppositely aligned. This result in the interdigitated arrangement of the alkyl chain of stearic acid molecules bonded to adenine molecules, and then keeps the lamella characteristic of the assembly of pure stearic acid assembly system. However, the dark trough made up of two –CH_3_ groups and two –COOH groups disappeared because of the inserted adenine molecules of SA-_s1_A_s5_-SA trimers. Thus, the inserted adenine in the middle of the lamella structure of stearic acid molecules can increase the distance along â and $\hat{b}$ vector direction. The unit cell was drawn out as indicated by a white rectangle in Figure 2c, and its parameters have good consistency with those of a = 6.43 ± 0.20 nm, b = 1.12 ± 0.20 nm, and ϕ = 89.8 ± 2° in HR-STM image (Figure 2b). Therefore, this further proves that adenine molecules were successfully introduced into the lamella-like configuration of the stearic acid molecules, and the hetero-complex of biological and chemical organic molecules composed of SA-A-SA had been fabricated.

## Conclusions

Straight-chain hydrocarbon and its derivatives can form well-aligned lamella structures, which not only supply a series of concise models for understanding the basic physical chemistry mechanism of adsorption and self-assembly on surface, but also provide available platform for the assembly of multi-component supramolecular structures with specific functionality (Ghijsens et al., 2013). In this work, the nucleobase adenine was mixed with fatty acid stearic acid to explore the formation of SA-A-SA hetero-complex linked through the intermolecular interaction of hydrogen-bonding. It is revealed that the interesting striped-shaped structure was formed and composed of SA-A-SA trimers with introducing adenine molecules. Meanwhile, the primary assembled characteristics of the lamella-shape of stearic acid and the chain-arranged of adenine were both kept because of the effective collaboration of the hydrogen-bonding of molecule-molecule and vdWs interaction of molecule-substrate. This research benefits the modification of existing assemblies for both chemists and biologists, and further provides a referential approach to explore the multi-component complexes through SAM method.

## Author Contributions

All authors listed have made a substantial, direct and intellectual contribution to the work, and approved it for publication.

### Conflict of Interest Statement

The authors declare that the research was conducted in the absence of any commercial or financial relationships that could be construed as a potential conflict of interest.
