# Supplementary material for: Fabrication of 2D Hetero-Complexes With Nucleic-Acid-Base Adenine and Fatty-Acid Stearic Acid at Liquid/Solid Interface
Source: Front Chem. 2019 Jul 25;7:513. doi: 10.3389/fchem.2019.00513 (PMC6669938; doi:10.3389/fchem.2019.00513)
Supplement: Supplementary file 1 [file Data_Sheet_1.pdf]

Supplementary Material

Fabrication of 2D Hetero-complexes with Nucleic-acid-base Adenine and Fatty-acid Stearic Acid at Liquid/solid Interface

Huiling Zhao[1,2], Qian Yang[2], Zegao Wang[2,3], Hang Zhao[4], Bo Liu[1]* Qianming Chen[4] and Mingdong Dong[2]*

[1] School of Physics and Electronics, Henan University, Kaifeng, 475004, China.

[2] Interdisciplinary Nanoscience Centre (iNANO), Sino-Danish Center for Education and Research (SDC), Aarhus University, DK-8000 Aarhus C, Denmark.

[3] College of Materials Science and Engineering, Sichuan University, Chengdu 610065, China

[4] State Key Laboratory of Oral Diseases, National Clinical Research Center for Oral Diseases, West China Hospital of Stomatology, Sichuan University, Chengdu, Sichuan 610041, China

*** Correspondence:**
Bo Liu and Mingdong Dong
boliu@henu.edu.cn; dong@inano.au.dk.

1.1 Supplementary Figures

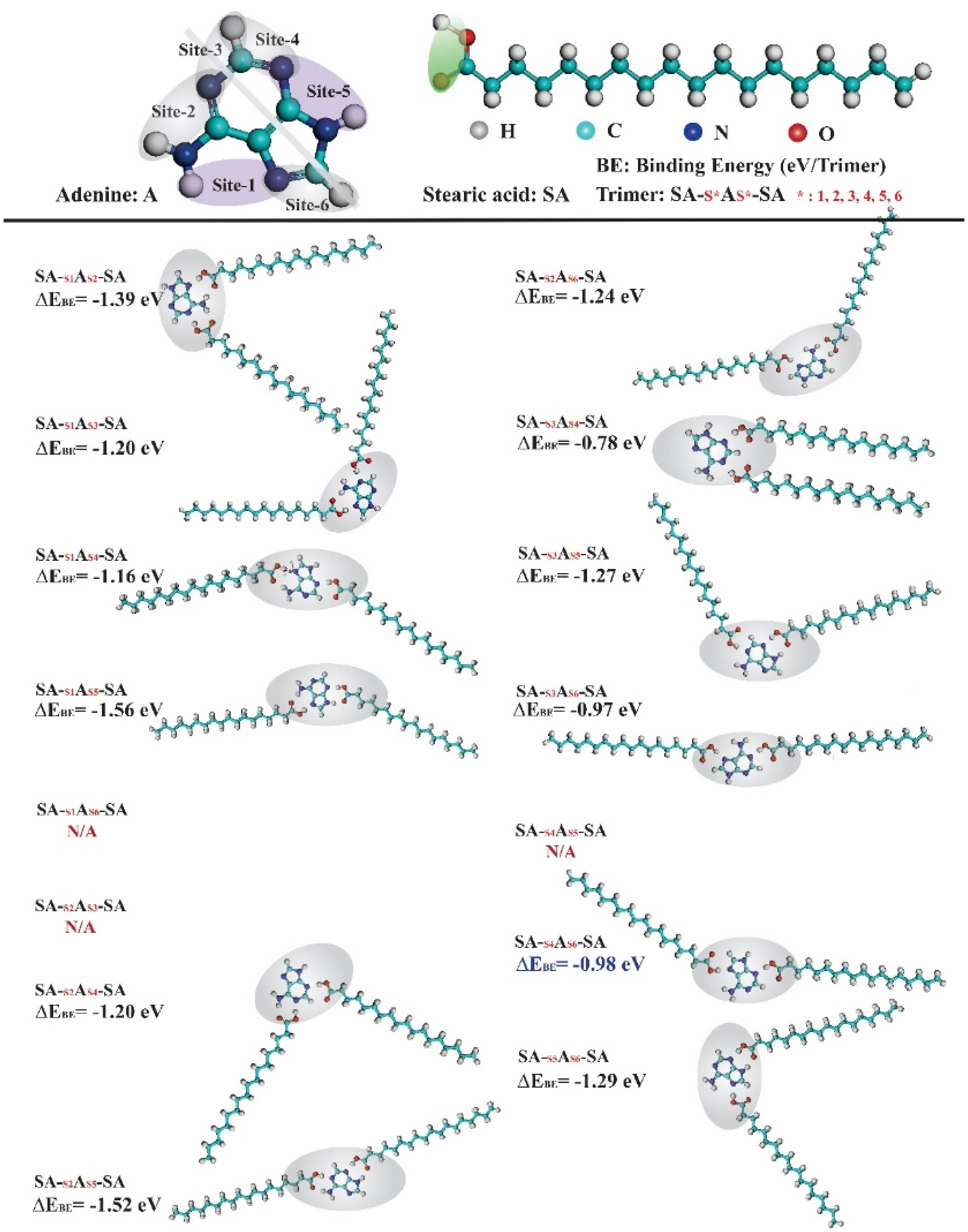

Supplementary Figure 1. Top images: Chemical structures of two kinds of assembled molecules: stearic acid (SA) and adenine (A). Down images: all models of SA-A-SA trimers and their corresponding binding energies derived from theoretical calculations.

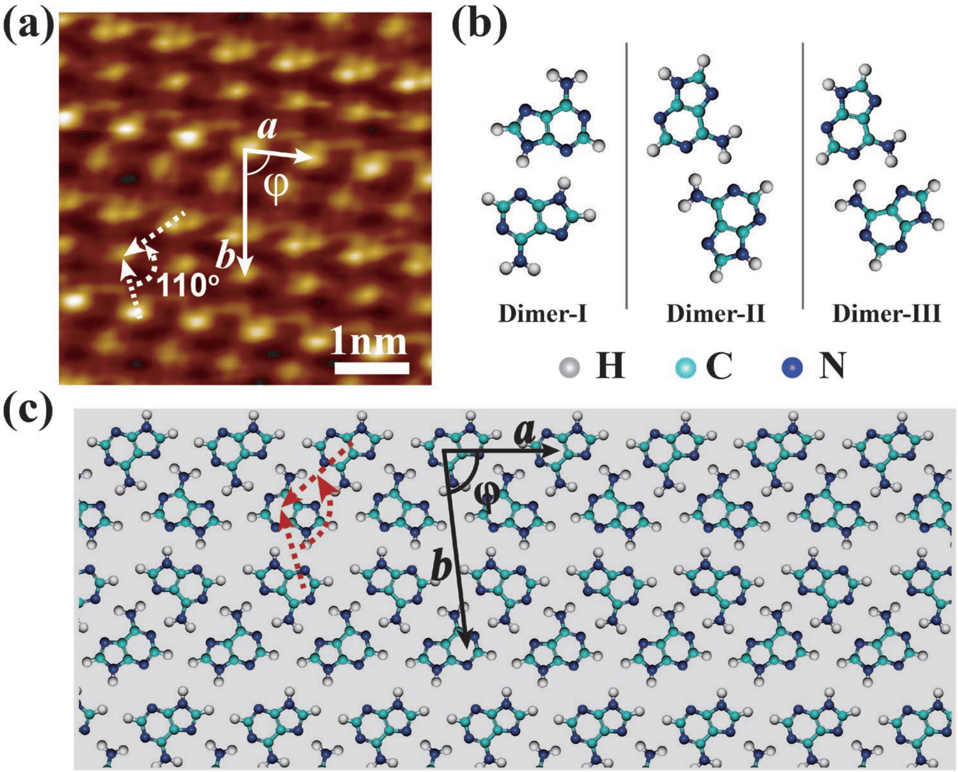

Supplementary Figure 2. (a) STM image of the assembled structure formed by pure adenine (A) system. (b) Three kinds of theoretical models for the A-A dimer formed by hydrogen-bonding interaction. (c) Theoretical model for the linear assembled structure originated from Dimer-III.

	**a**	**b**	**φ**	**Trimer angle**
**SA-SA**	4.43±0.20 nm	0.82±0.20 nm	80.0°±2°	**180°**
**A-A**	0.80±0.1 nm	2.20± 0.2 nm	76.0°±2°	**110°**
**SA-A-SA**	6.43±0.20 nm	1.12±0.20 nm	89.8°±2°	**140°**

Supplementary Table 1. The parameter values for three kinds of assembled unit cells marked in their STM images and corresponding theoretical models in Figures 1(b)(c), Figures 2(b)(c) and Supplementary Figures 2(b)(c).
